# Physical activity in wheelchair-using youth with spina bifida: an observational study

**DOI:** 10.1186/s12984-018-0464-x

**Published:** 2019-01-14

**Authors:** Manon A. T. Bloemen, Rita J. G. van den Berg-Emons, Matthijs Tuijt, Carla F. J. Nooijen, Tim Takken, Frank J. G. Backx, Marleen Vos, Janke F. de Groot

**Affiliations:** 1 Research Group Lifestyle and Health, HU University of Applied Sciences Utrecht, Utrecht, The Netherlands; 2 Department of Rehabilitation Medicine, Erasmus University Medical Center, Rotterdam, the Netherlands; 3 Research Group Human Movement and Adaptation, HU University of Applied Sciences Utrecht, Utrecht, The Netherlands; 4 Department of Public Health, Karolinska Institutet, Stockholm, Sweden; 5 Child Development and Exercise Center, Wilhelmina Children’s Hospital, University Medical Center Utrecht, Utrecht, The Netherlands; 6 Department of Rehabilitation, Physical Therapy Science and Sports, University Medical Center Utrecht, Utrecht, The Netherlands; 7 Quality and Organization of Care, Netherlands Institute for Health Services Research, Utrecht, The Netherlands

**Keywords:** Spinal Dysraphism, Children, Adolescents, Youth, Wheelchairs, Physical activity

## Abstract

**Background:**

Even though typically developing youth are already at risk for physical inactivity, youth with spina bifida may be even at higher risk as a consequence of their reduced mobility. No objective data is available for youth with spina bifida who use a manual wheelchair, so the seriousness of the problem is unknown. The purpose of this observational study was to quantify physical activity in wheelchair-using youth with spina bifida and evaluate the intensity of activities.

**Methods:**

Fifty-three children and adolescents (5–19 years) with spina bifida who use a manual wheelchair for daily life, long distances or sports were included. To assess time spent in several types of activities VitaMove data of 34 participants were used and were presented as time spent sedentary and time spent physically active. This was compared to reference data of typically developing youth. To assess time spent in several intensities Actiheart data of 36 participants were used. The intensities were categorized according to the American College of Sports Medicine, ranging from very light intensity to near to maximal intensity. Data of 25 participants were used to combine type of activity and intensity.

**Results:**

Children and adolescents with spina bifida who use a manual wheelchair were more sedentary (94.3% versus 78.0% per 24 h, *p* < 0.000) and less physically active (5.0% versus 12.2% per 24 h, *p* < 0.000) compared to typically developing peers. Physical activity during weekend days was worse compared to school days; 19% met the Guidelines of Physical Activity during school days and 8% during weekend days. The intensities per activity varied extensively between participants.

**Conclusions:**

Children and adolescents with spina bifida who use a manual wheelchair are less physically active and more sedentary than typically developing youth. The physical activity levels on school days seem to be more favorable than the physical activity levels on a weekend day. The low levels of physical activity need our attention in pediatric rehabilitation practice. The different intensities during activities indicate the importance of individually tailored assessments and interventions.

## Background

Even though typically developing youth are already at risk for physical inactivity, youth with spina bifida (SB) may be even at higher risk as a consequence of their reduced mobility or time spent in the wheelchair [1–3]. A lack of physical activity (PA) in this population may lead to secondary complications that have major negative health effects such as obesity, hypertension, orthopedic concerns, coronary heart disease, and type 2 diabetes. A recent review indeed showed that adolescents and adults with SB have higher rates of obesity, body fat and cardiovascular disease risks compared to the unimpaired reference population [4, 5].

Different concepts can be considered when measuring PA, such as time spent in different activities but also the physiologic response of the body resulting in elevated heart rates (HRs), representing the intensity of PA [6, 7]. Information about the different activities will give insight into whether children for example spend a large amount of time sedentary or physically active (f.e. self-propel their wheelchair instead of being pushed or use (hand)biking as active transport). The intensity of PA is important because of its expected relationship with aerobic fitness and its long term health benefits. It provides insight into whether children comply to international guidelines for PA (> 60 min moderate to vigorous intensity of which 30 min > vigorous intensity) [8, 9].

A study in ambulatory youth with SB showed decreased self-reported PA compared to typically developing peers [10]. Accelerometry-based evidence is only available for adolescents and young adults (mean age 21 years) with SB, overall showing an elevated time spent sedentary and reduced time spent physically active, with the small subgroup of wheelchair-using participants as least physically active [11]. To our knowledge there is no accelerometry-based PA evidence available for wheelchair-using youth with SB. Moreover, information about objectively measured intensity of daily PA is also lacking for this population. Even though we expect that wheelchair-using youth with SB spend more time sedentary and less time physically active compared to typically developing peers and that the majority does not comply to guidelines for PA, we do not have any objective evidence to truly understand the seriousness of the problem of physical inactivity in this young population.

Combining type of activity and intensity is interesting as it provides information about at which intensities certain activities are performed during normal daily life; this might be different compared to typically developing peers because of the disability [12, 13]. While it could seem that wheelchair-using youth are less active as defined by time spent sedentary or physically active, the intensity level could show other results. Therefore, the aims of this study were:

1. To describe time spent sedentary and physically active of wheelchair-using youth with SB and compare this with typically developing peers;
2. To describe the intensity of daily PA and the compliance to guidelines of PA;
3. To describe the intensity of different types of activities during daily life.

## Methods

### Participants

This observational study is part of the “*Let’s Ride…study*”, evaluating physical fitness and physical behavior in wheelchair-using youth with SB. Participants were recruited in the Netherlands and were included if they were diagnosed with SB, 5–18 years of age during enrolment, used a manual wheelchair during daily life, for long distances or for sports participation and were able to follow test instructions. Participants were excluded if they had any events that might intervene with the testing outcomes. All parents and participants aged 12 years and older signed informed consent. The Medical Ethics Committee of the University Medical Center Utrecht approved the study procedures (number 11–557) [14–16].

### Demographic and morphologic parameters

The participants visited our lab to record age, gender, type of SB, lesion level, sport activities and type of wheelchair by a standard questionnaire. Body mass was measured using an electronic wheelchair scale (Kern MWS-300K100M, KERN & SOHN GmbH, Balingen, Germany) and height was measured using a non-stretchable tape while seated using the arm span length (middle finger-tip to middle finger-tip) as recommended in wheelchair-using youth, due to the presence of contractures when lying supine [17]. The body mass index (BMI, body mass divided by the square of length) was adjusted with × 0.95 for mid-lumbar lesions and with × 0.90 for high lumbar/thoracic lesions [17].

### Equipment for measuring type of activity and intensity

**Fig. 1:**
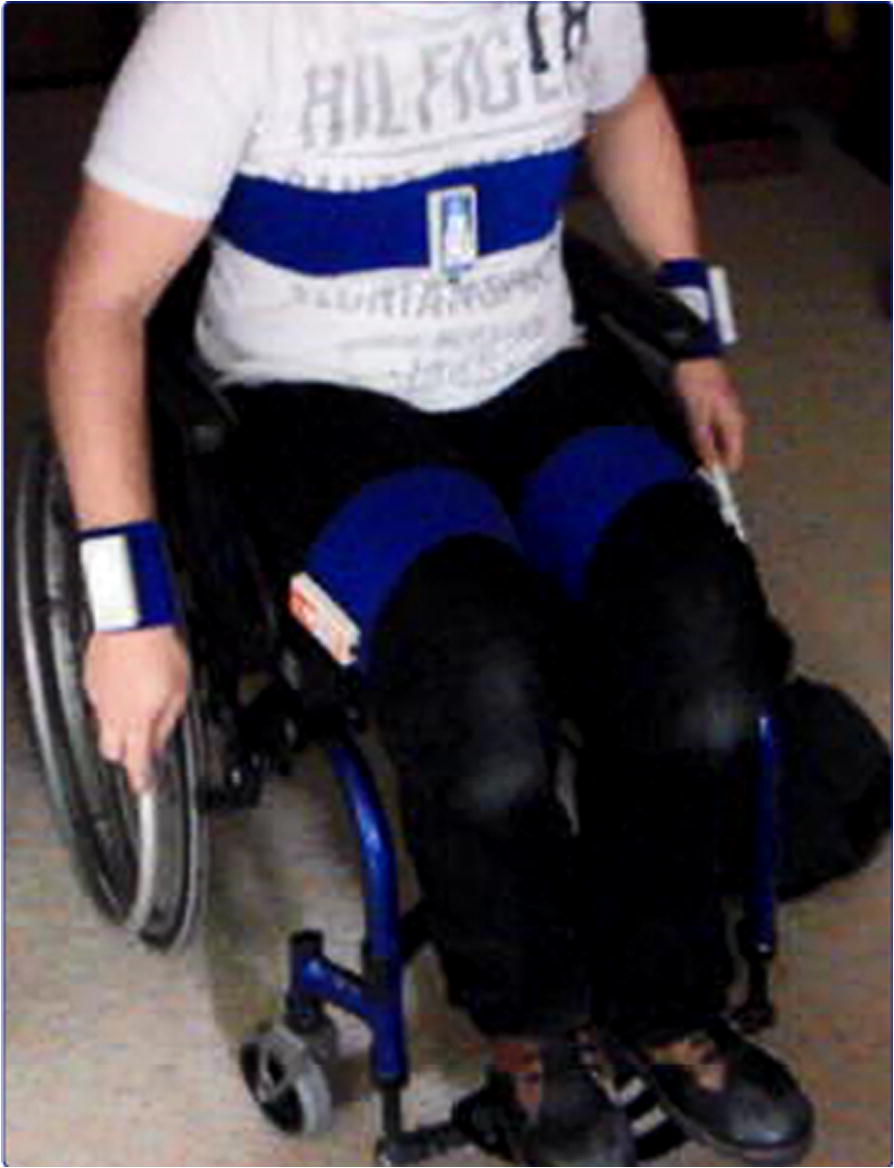
VitaMove activity monitor placed on an adolescent who is wheelchair-using and able to walk

The VitaMove (2M Engineering, Veldhoven, the Netherlands) was used for measuring time spent in different types of activities. The VitaMove is a monitoring system with wireless body-fixed accelerometers (Freescale MMA7260Q, Denver, USA) and is highly valid for measuring mobility-related activities in wheelchair-using youth as well as in able-bodied people (Fig. 1) [18–20].

For non-ambulatory participants, the system consists of three recorders: one recorder is placed on the sternum and a recorder is placed on each wrist. The following activities can be distinguished: lying, sitting, wheeling, handbiking and non-cyclic moving (=moving arms while sitting but not wheeling). Participants who were both walking *and* wheelchair-using wore two additional recorders, one on each thigh, to additionally distinguish standing, walking, running, and biking. Accelerometer signals from each recorder were sampled with a frequency of 128 Hz and stored digitally on a micro Secure Digital memory card. Data from all cards were uploaded to a computer for kinematic analysis using VitaScore Software (VitaScore BV, Gemert, the Netherlands). The analysis was an automated process consisting of three parts: (1) feature processing, (2) activity detection and (3) post-processing. Detailed descriptions of the configuration and analysis have been described elsewhere [18, 19].

Outcomes of the Vitamove included time spent in different activities expressed as a % of wear time. Sitting and lying were clustered as time spent sedentary. Walking, running, wheeling, (hand)biking and non-cyclic moving were clustered as time spent physically active. Standing was presented separately.

**Fig. 2:**
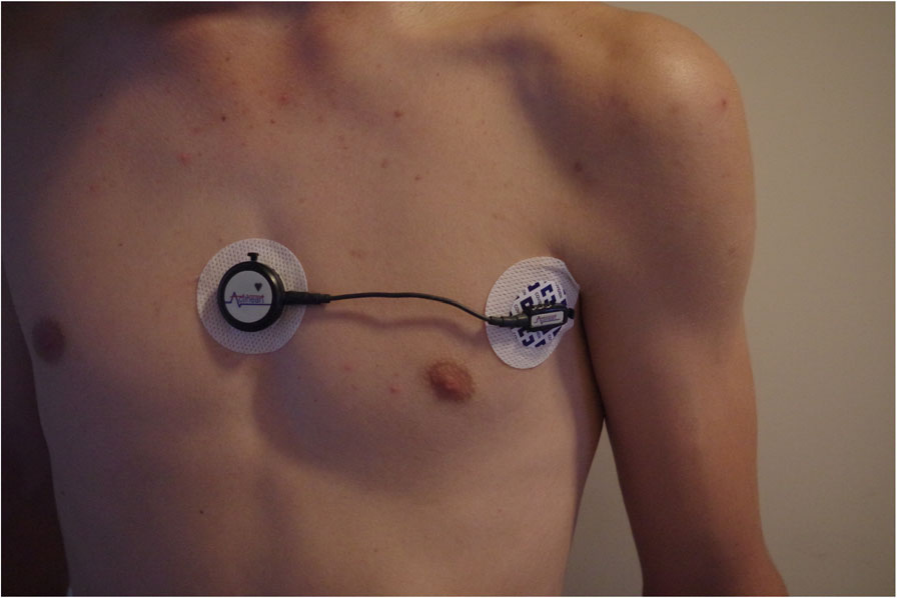
Actiheart placed on a participant

The Actiheart (CamNtech Ltd., Papworth Everard, United Kingdom) was used for measuring the intensity of PA. It is a highly valid device for measuring HR (every 30 s) and was attached to the chest by electrocardiogram electrodes (H99SG, Kendall, Covidien, Ireland) (Fig. 2) [21]. The Actiheart measures the ECG signal, which is electronically amplified by a factor of 900 with a frequency response of 10 to 35 Hz. From the resulting ECG signal, R-R interval durations are calculated, and averaged over epochs of 30 s. The averaged R-R interval duration is subsequently converted to beats per minute (bpm) and stored in memory [21].

As a measure of intensity, the heart rate reserve (HRR) was determined from the registered HRs by the Actiheart, using the following formula [23]:

$$ \mathrm{HRR}=\left({\mathrm{HR}}_{\mathrm{measured}\ \mathrm{by}\ \mathrm{actiheart}}-{\mathrm{HR}}_{\mathrm{rest}}\right)/\left({\mathrm{HR}}_{\mathrm{peak}}-{\mathrm{HR}}_{\mathrm{rest}}\right)\times 100\% $$

For the “*Let’s Ride… study”*, both the Shuttle Ride Test and the Graded Wheelchair Propulsion Test were performed to determine HR_peak_; these are valid and reliable maximal exercise tests for wheelchair-using youth with SB [14, 16]. HR_rest_ was measured after sitting still for 10 min. If either a higher HR_peak_ or a lower HR_rest_ was measured by the Actiheart in daily life, the latter values were used [24]. The HRRs were classified into five intensity zones according to the American College of Sports Medicine (ACSM) with 0–30% HRR classified as very light, 30–40% as light, 40–60% as moderate, 60–90% as vigorous and > 90% as near to maximal [25]. To control for differences in wear time, both minutes per day and % of wear time were determined.

### Protocol

Both devices were distributed to and collected from the participants in person. Participants were asked to wear both devices simultaneously for two school days and one weekend day from the moment they got dressed until they went to bed, except during bathing and swimming. We provided clear descriptions to the children and parents about how to remove and apply the devices. We used written information, photos and real life instruction. In case of any questions, they could always contact the researcher. The participants and their parents were also asked to keep an activity diary so we could correct for swimming, check for peculiarities in the data and check the wear time. To avoid measurement bias, we instructed participants to continue ordinary life. A minimum duration of 1 day and a minimum wear time of 8 h per day (derived from the devices and checked using the diaries) was required to be included in this study [22]. Data were excluded if participants were ill during recording days.

### Analyses

To address the time spent sedentary, the time spent physically active and the intensity of daily PA, SPSS statistics 23.0 (International Business Machines Corp) was used. Histograms, QQ-plots and the Shapiro Wilk test showed that data of the Vitamove and Actiheart separately were not normally distributed so we used non-parametric descriptive statistics. Wilcoxon Signed Rank tests showed no differences (*p* > 0.241 for data of the VitaMove and *p* > 0.584 for data of the Actiheart) between the first and second school day, justifying the use of data when only one school day was available. When data of two school days were available, data were averaged. Furthermore, the Wilcoxon Signed Rank test showed significant differences (Table 4 and Table 5) between school days and weekend days, so the school days and weekend days were analyzed separately.

For comparison of the VitaMove data with typically developing peers, reference data of 20 typically developing youths (age and gender matched) were used who had worn the VitaMove during two school days (48-h measurement). These data were available as a % of 24 h from previous studies at the department of Rehabilitation Medicine at Erasmus University Medical Center Rotterdam, therefore we also expressed our data as a % of 24 h for this comparison [26]. Differences were analyzed using linear regression correcting for gender and age.

To address the intensities of different types of activities during daily life, data of the VitaMove and data of the Actiheart were combined using MatLab (MatLab, R2014b, The MathWorks Inc., Natick, MA, USA). We upsampled the data of the Actiheart so the 30-s intervals of the Actiheart could be combined with the 1-s intervals of the VitaMove. Parametric descriptive statistics were used to analyze time spent (both % of wear time and minutes) in at least moderate PA (> 40% HRR) and time spent in at least vigorous PA (> 60% HRR) per type of activity.

## Results

A total of 53 wheelchair-using youths with SB participated in the *Let’s Ride…study*. VitaMove data of 34 participants could be used for analyzing type of activities and Actiheart data of 36 participants could be used for analyzing intensity of PA. For intensity of different activities, data of 25 participants could be combined (VitaMove and Actiheart) (Table 1). Missing data were caused by not properly functioning of devices, wear time less than 8 h (f.e. because of skin irritation) or illness (Table 2). While compliance of wearing the Actiheart was higher than compliance of wearing the VitaMove, we did experience some data loss with the Actiheart in the beginning of the study because the Actiheart was not able to properly record the signals. By using the electrocardiogram electrodes type H99SG (Kendall, Covidien, Ireland), the Actiheart was better able to pick up the signals compared to using other electrocardiogram electrodes. There were no significant differences between characteristics of participants with VitaMove / Actiheart data and missing data, assuming that the missing data was random.

**Table 1:** Characteristics of the participants

	VitaMove	Actiheart	VitaMove - Actiheart
	*N* = 34	*N* = 36	*N* = 25
	Mean (SD)	Mean (SD)	Mean (SD)
Age (years;months)	13.7 (3.2)	13.5 (3.6)	13.4 (3.3)
Body mass (kg)	52.8 (18.1)	49.7 (19.5)	53.4 (19.3)
Height (cm)	159.1 (19.5)	155.5 (21.4)	158.9 (21.2)
Body Mass Index (kg/m^2^)	23.9 (6.3)	23.2 (7.4)	24.1 (7.2)
• Normal weight (n)	15	20	11
• Overweight (n)	9	7	6
• Obese (n)	10	9	8
Weight wheelchair (kg)	19.6 (6.7)	19.1 (5.8)	19.5 (5.9)
Heartrate rest (beat per minute)	na	76 (9)	77 (8)
Heartrate peak (beats per minute)	na	189 (15)	189 (13)
Heartrate reserve	na	113 (17)	113 (16)
	N	N	N
Gender (boys/girls)	20/14	21/15	15/10
Sports (no/1xweek/2xweek/3xweek)	7/14/9/4	6/17/9/4	5/11/6/3
Type (aperta/occulta)	33/1	33/3	24/1
Level of lesion			
• Thoracic	5	6	2
• Lumbar	29	29	23
• Sacral	0	1	0
Ambulation level			
• Community ambulatory	2	4	2
• Household ambulatory	3	6	3
• Therapeutic ambulatory	3	2	1
• Non ambulatory	26	24	19

*N* number of participants, *SD* standard deviation, *kg* kilogram, *cm* centimeter, *m* meter, *na* not applicable

**Table 2:** Overview of missing data

	VitaMove	ActiHeart
Device did not function properly (Number of participants)	7 (all days)	9 (all days)
	2 (both school days)	2 (both school days)
	1 (1 school day)	5 (1 school day)
		1 (1 school + weekend day)
Stopped wearing the device because of irritation of the skin (Number of participants)	1 (all days)	1 (all 3 days)
	1 (weekend day)	1 (1 school day)
		1 (weekend day)
Wear time < 8 h (Number of participants)	1 (all days)	1 (1 school + weekend day)
	4 (1 school day)	4 (weekend day)
	1 (1 school + weekend day)	
	1 (weekend day)	
Holiday or due to illness (Number of participants)	3 (all days)	3 (all days)

**Fig. 3:**
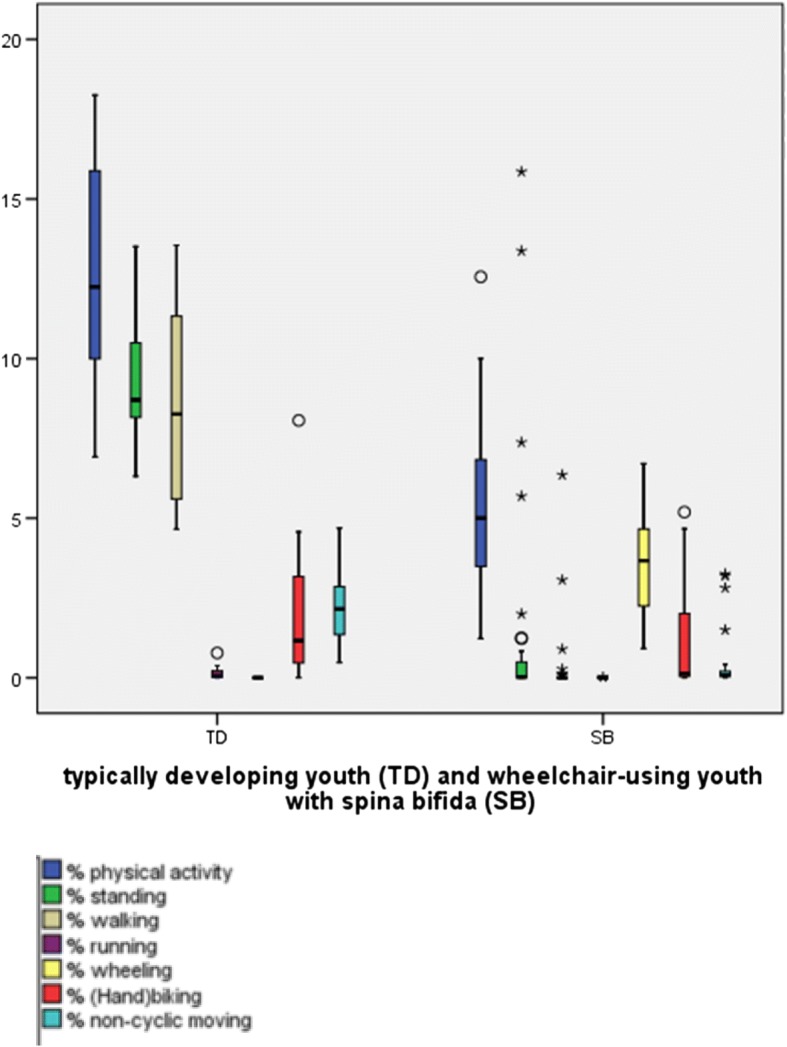
Type of physical activity presented in % of 24 h

**Fig. 4:**
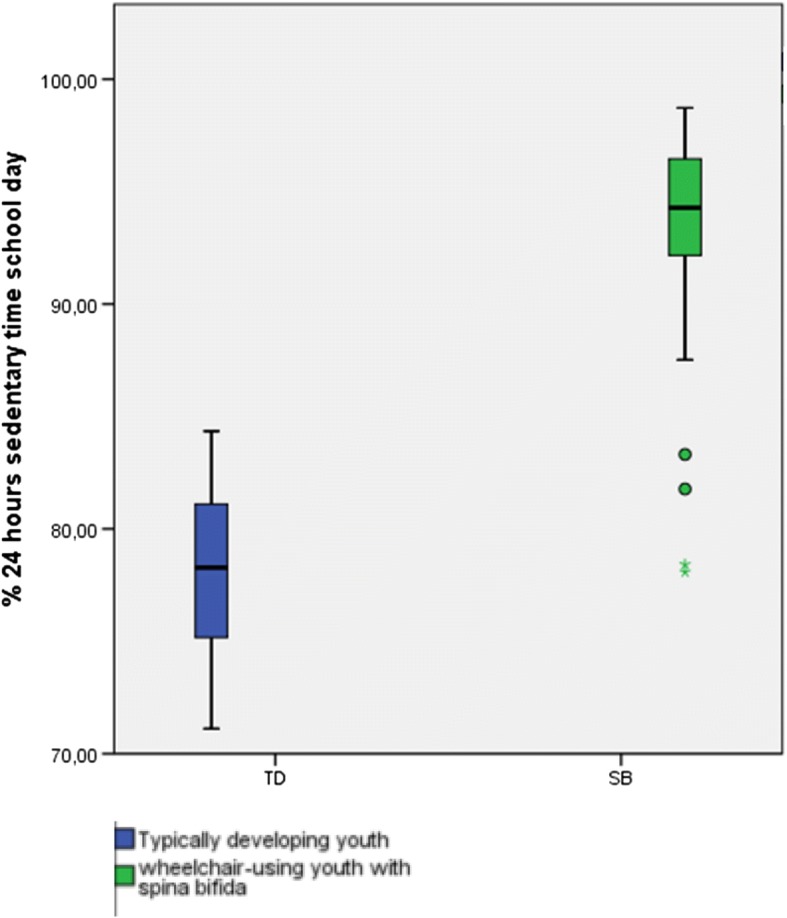
Sedentary activity presented in % of 24 h

**Fig. 5:**
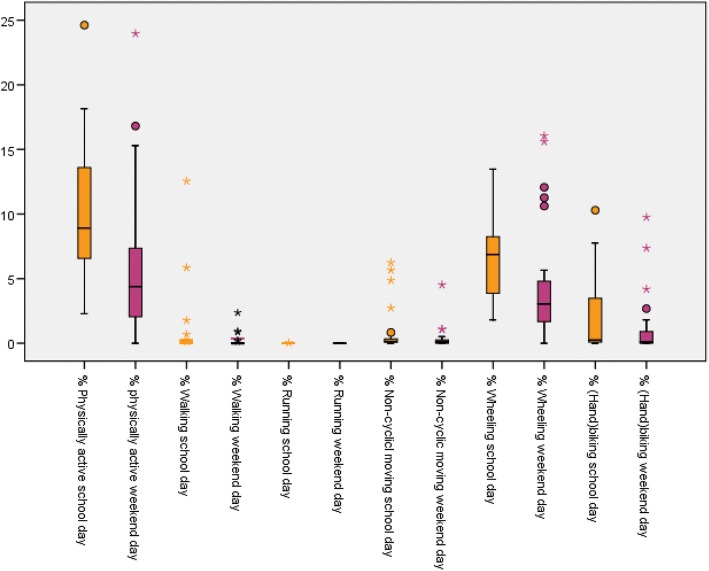
Type of physical activity presented in % wear time for school days and weekend days separately

**Fig. 6:**
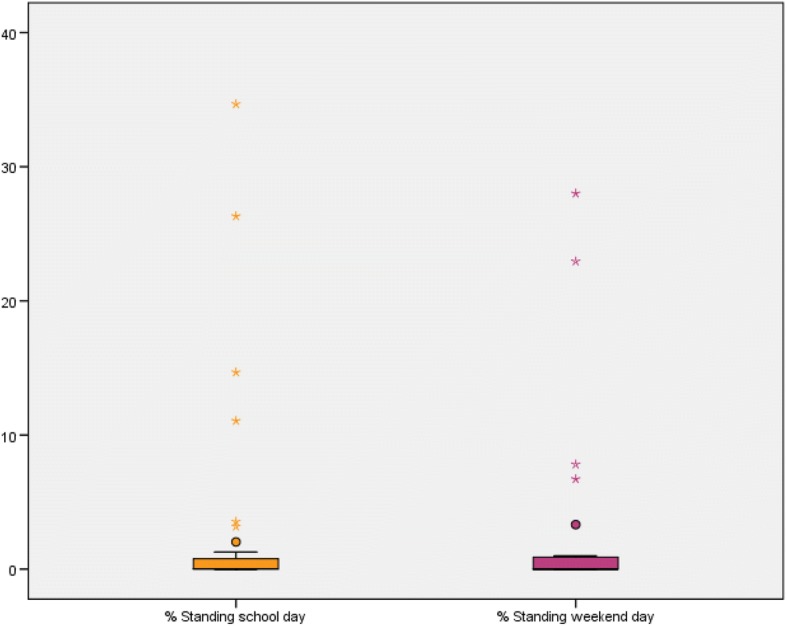
Standing presented in % wear time for school days and weekend days separately

**Fig. 7:**
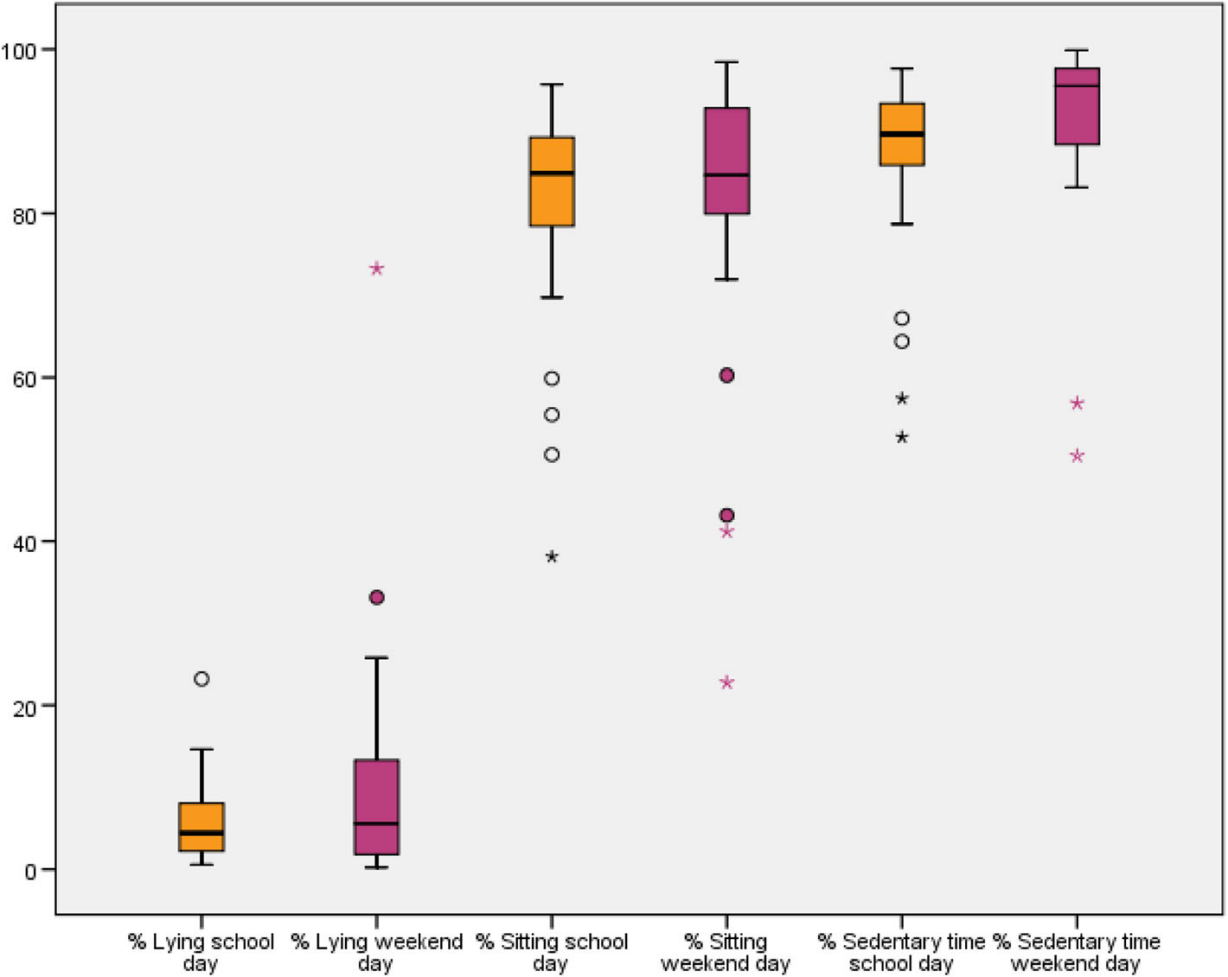
Sedentary activities presented in % wear time for school days and weekend days separately

**Fig. 8:**
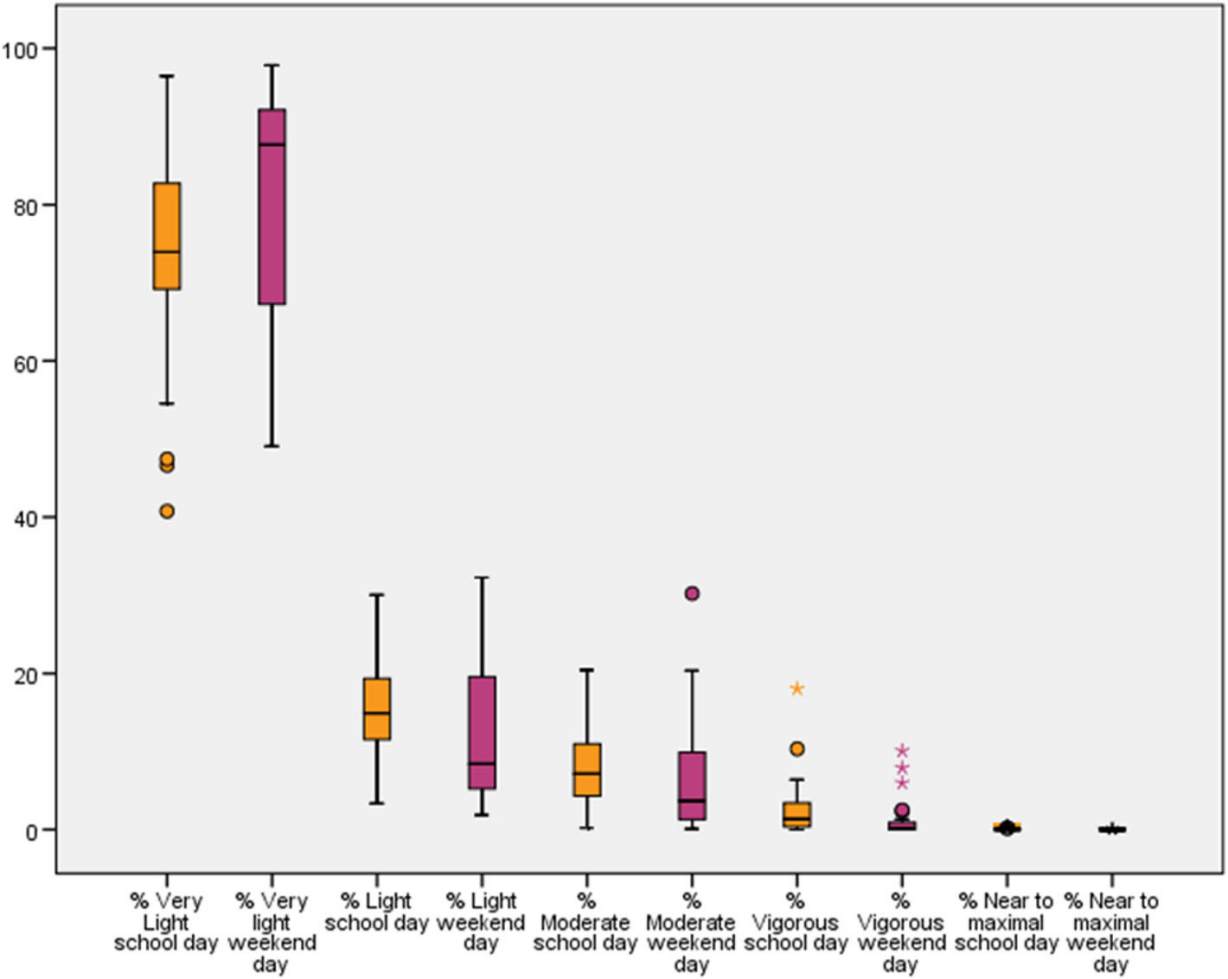
Intensities presented in % wear time for school days and weekend days separately

To address our first research aim about the description of the time spent sedentary and the time spent physically active, we presented the results in Tables 3 and 4 and Figs. 3, 4, 5, 6, 7 and 8. Wheelchair-using youth with SB spent 90% of the wear time (IQR 7%) sitting or lying during a school day compared to 96% (IQR 10%) during a weekend day, which is significantly different (*p* = 0.007). Furthermore, they spent significantly (*p* = 0.003) more time physically active during a school day (median 9% of the wear time, IQR 7%) compared to a weekend day (median 4% of the wear time, IQR 6%).

**Table 3:** Percentage of time spent in different types of activities on a school day, comparing wheelchair-using youth with SB to typically developing peers

	Youth with SB	Youth who is typically developing	Difference	*P* ^c^
Characteristics				
Number of participants	32	20		
Age (years) mean (SD)	13.7 (3.2)	13.8 (2.9)	−0.1	0.939
Gender (boys/girls)	20/14	10 / 10		0.580
Weight (kg) mean (SD)	52.8 (18.1)	45.7 (14.3) (*n* = 10)	7.1	0.258
Height (cm) mean (SD)	159.1 (19.5)	158.5 (14.5) (n = 10)	0.6	0.929
% of time physically active^a^	5.0 (3.5; 1.2–12.6)	12.2 (6.1; 6.9–18.3)	−7.2	0.000
Median (IQR; min-max)				
• Walking	0.0 (0.0; 0.0–6.4)	8.3 (6.2; 4.7–13.6)		
• Running	0.0 (0.0; 0.0–0.0)	0.1 (0.2; 0.0–0.8)		
• Wheeling	3.7 (2.5; 0.9–6.7)	0.0 (0.0; 0.0–0.0)		
• (Hand)biking	0.1 (2.1; 0.0–5.2)	1.2 (3.0; 0.0–8.1)		
• non-cycling movement	0.1 (0.2; 0.0–3.3)	2.2 (1.5; 0.5–4.7)		
% of time sedentary^b^ (sitting and lying)	94.3 (4.3; 78.1–98.7)	78.3 (6.3; 71.1–84.3)	16.3	0.000
Median (IQR; min-max)				
% Standing	0.0 (0.7; 0.0–15.9)	8.7 (2.4; 6.3–13.5)	−8.7	
Median (IQR)				

*SB* spina bifida, *SD* standard deviation, *kg* kilogram, *cm* centimeter, *IQR* Interquartile range, *min* minimum, *max* maximum

^a^Time spent physically active = total duration of walking, running, wheeling, (hand)biking and non-cyclic moving, as a % of 24 h

^b^Time spent sedentary = total duration of sitting and lying, as a % of 24 h

^c^Difference in characteristics between participants with SB and typically developing children was tested with a two sample t-test (age, weight, height) and chi-square (gender). Differences in time spent physically active and time spent sedentary were analyzed with regression analyses corrected for age and gender

**Table 4:** Duration of the types of activities in wheelchair-using youth with SB, separately presented for a school day and a weekend day

	School day Median (IQR; minimum-maximum)	Weekend day Median (IQR; minimum-maximum)	*P* ^d^
Wear time VitaMove^a^	13.2 (1.6)	10.9 (1.9)	0.000
Time spent physically active^b^	9 (7; 2–24)	4 (6; 0–24)	0.003
• Walking	0 (0; 0–13)	0 (0; 0–2)	
• Running	0 (0; 0–0)	0 (0; 0–0)	
• Non-cyclic moving	0 (0; 0–6)	0 (0; 0–5)	
• Wheeling	7 (5; 2–13)	3 (4; 0–16)	
• (Hand)biking	0 (3; 0–10)	0 (1; 0–10)	
Time spent sedentary^c^	90 (7; 53–98)	96 (10; 50–100)	0.007
• Sitting	84 (11; 38–96)	85 (15; 23–98)	
• Lying	4 (6; 1–23)	6 (12; 0–73)	
Standing	0 (1; 0–35)	0 (1; 0–28)	

Type of activities are presented as % of wear time (median, interquartile range and minimum-maximum)

^a^Wear time is total wear time in hours, presented as mean (standard deviation)

^b^Time spent physically active = total duration of walking, running, non-cyclic movement, wheeling and (hand)biking, presented as a % of wear time

^c^Time spent sedentary = total duration of sitting and lying, presented as a % of wear time

^d^Differences between a school day and weekend day for wear time was tested with the paired samples t-test. Differences between a school day and weekend day for time spent physically active and time spent sedentary were tested with the Wilcoxon Signed Rank test

Compared to typically developing peers, wheelchair-using participants with SB spent a significantly higher amount of time sedentary (94% per 24 h versus 78% per 24 h, *p* < 0.000) and a significantly lower amount of time physically active (5% per 24 h versus 12% per 24 h, *p* < 0.000) on a school day (Table 4, Figs. 3 and 4). This corresponds with approximately 72 min physically active on a school day for wheelchair-using youth with SB compared to 175 min for typically developing peers.

To address our second research aim about the intensity of daily PA and the compliance to the guidelines of PA (research aim 2), we presented our results in Table 5 and Fig. 8. Looking at the Guidelines of PA from the ACSM, 19% of the participants (three community ambulatory, one household ambulatory, three non-ambulatory) met these guidelines during a school day and 8% (two community ambulatory, one non-ambulatory) during a weekend day [8]. The participants seemed to spent a higher amount of time in very light physical activity (*p* = 0.045) during a weekend day compared to a school day and a higher amount of time in light (*p* = 0.032), moderate (*p* = 0.086), vigorous (*p* = 0.010) and near to maximal (*p* = 0.002) physical activity during a school day compared to a weekend day. The significance of these results should be interpreted with caution because of the multiple tests that were performed.

**Table 5:** Intensity of PA for wheelchair-youth with SB, separately presented for school days and weekend days

	Total Minutes Median (IQR; minimum-maximum)			% Wear time Median (IQR; minimum-maximum)		
	School day	Weekend day	*P*	School day	Weekend day	*P*
Wear time	761 (117)	628 (140)	0.000			
Very light (0–30%)	575 (180; 255–772)	500 (145; 304–702)	0.010	73.96 (14.37; 40.76–96.44)	87.71 (25.90; 49.1–97.9)	0.045
Light (30–40%)	110 (57; 24–214)	56 (100; 10–206)	0.001	14.91 (8.29; 3.36–30.03)	8.43 (15.50; 1.9–32.2)	0.032
Moderate (40–60%)	55 (55; 2–146)	20 (76; 1–207)	0.027	7.15 (7.83; 0.21–20.45)	3.66 (9.40; 0.1–30.2)	0.086
Vigorous (60–90%)	10 (25; 0–104)	1 (7; 0–65)	0.005	1.35 (3.43; 0.00–18.05)	0.15 (0.90; 0.0–10.1)	0.010
Near-max to maximal (> 90%)	0 (1; 0–5)	0 (0; 0–1)	0.001	0.03 (0.11; 0.00–0.61)	0.00 (0.00; 0.0–0.2)	0.002

*IQR* Interquartile range

**Fig. 9:**
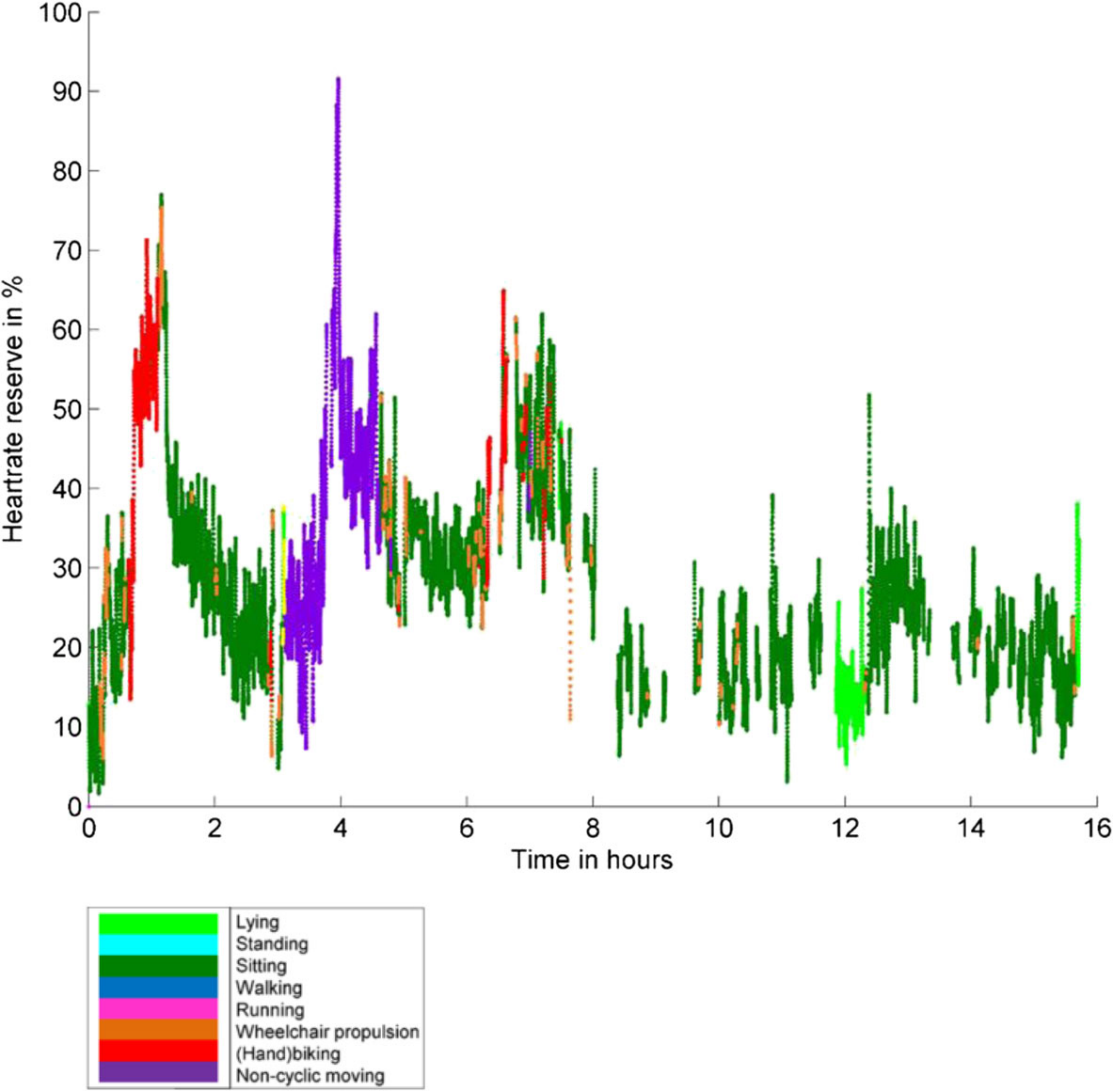
The % of HRR for the several types of activity performed on a school day by a wheelchair-using adolescent with SB. Unfortunately, some of the time series contain gaps due to either loss of signal, bathing, or swimming

**Fig. 10:**
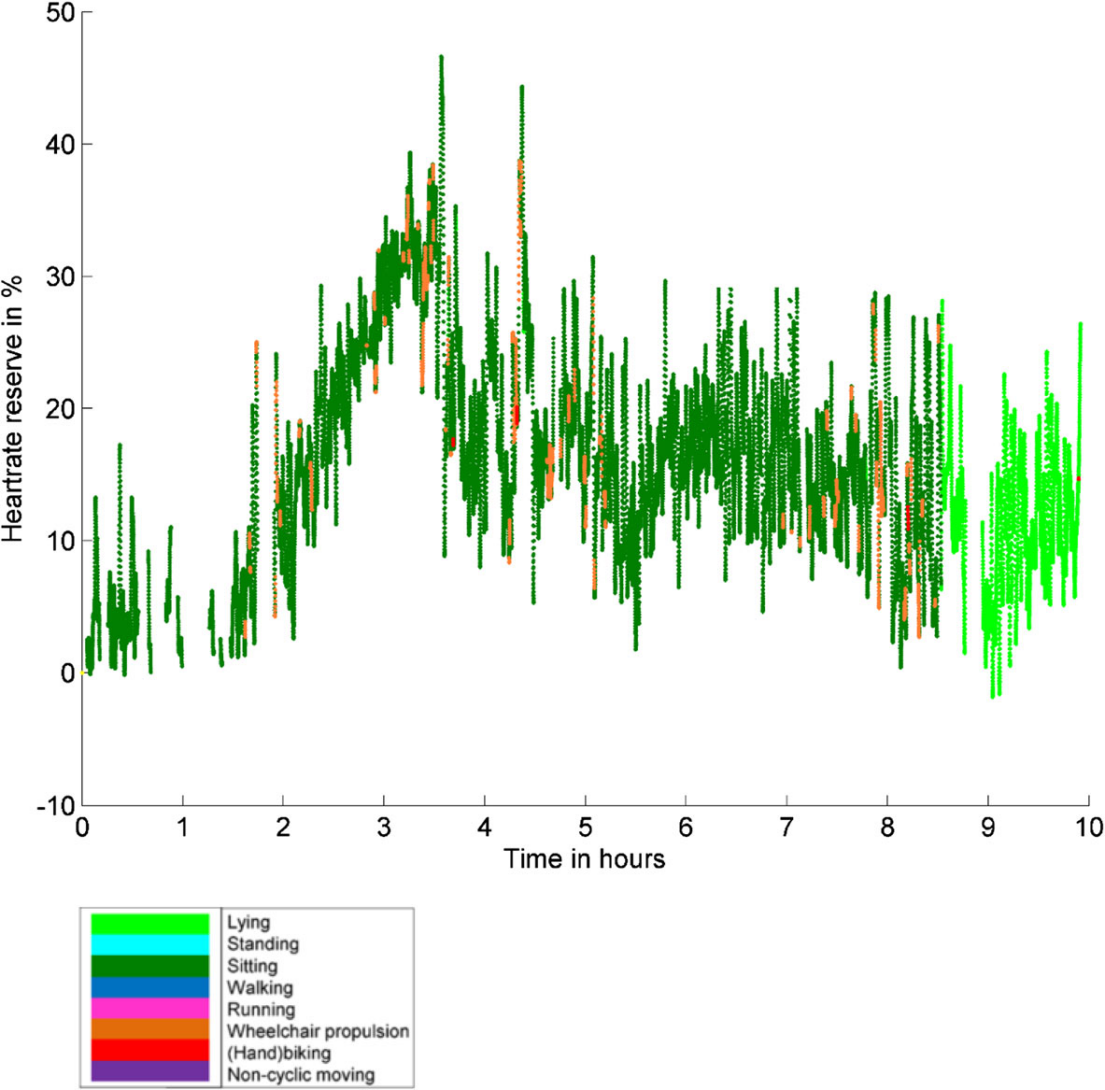
The % of HRR for the several types of activity performed on a weekend day by a wheelchair-using adolescent with SB. Unfortunately, some of the time series contain gaps due to either loss of signal, bathing, or swimming

To address our third research aim about the description of the intensity of different types of activities during daily life, we presented our results in Table 6. Looking at the mean intensities of the activities, lying, sitting and non-cyclic moving were mostly performed at a very light intensity level. Standing, wheeling and hand-biking were mostly performed at a light intensity level and walking was mostly performed at a vigorous intensity level. However, the ranges of the intensities per activity varied extensively, for example the intensity of wheeling varied from 0 to 98% and thus varied from a very light intensity level to a near to maximal intensity level. An example of the intensities of different activities for a wheelchair-using adolescent during a school day and weekend day is presented in Figs. 9 and 10; it clearly illustrates the differences in type of activities and intensity during a school day and a weekend day.

**Table 6:** Intensity of the different activities

	Lying	Sitting	Standing	Walking	Wheeling	(Hand)biking	Non-cyclic moving
%HRR^a^	22 (9; 0–76)	22 (10; 0–99)	36 (12; 0–86)	44 (12; 3–82)	33 (12; 0–98)	32 (9; 0–100)	29 (9; 5–71)
Minutes > 40% HRR^b^	0 (1; 0–7)	24 (27; 8–118)	19 (23; 9–37)	3 (25; 0–37)	6 (9; 0–64)	0 (7; 0–30)	0 (1; 0–19)
% of wear time > 40% HRR^b^	0 (0; 0–1)	4 (4; 1–15)	3 (3; 1–6)	1 (4; 0–6)	1 (1; 0–8)	0 (1; 0–4)	0 (0; 0–2)
Minutes > 60% HRR^b^	0 (0; 0–1)	2 (5; 0–14)	3 (9; 0–17)	1 (19; 0–23)	1 (2; 0–32)	0 (2; 0–16)	0 (0; 0–3)
% of wear time > 60% HRR^b^	0 (0; 0–0)	0 (1; 0–2)	0 (1; 0–3)	0 (2: 0–4)	0 (0; 0–4)	0 (0; 0–2)	0 (0; 0–0)

^a^% HRR presented as mean (standard deviation; minimum - maximum)

^b^Minutes / % of time > 40% HRR and Minutes / % of time > 60% HRR presented as median (interquartile range; minimum – maximum)

## Discussion

This study reports on objectively measured daily PA results of wheelchair-using youth with SB. As expected, they spent more time sedentary and less time physically active compared to typically developing peers. It was striking to learn though that typically developing peers were about 2.5 times more physically active. In addition, only 8–19% of our participants met the PA intensity guideline [8]. This indicates that, in general, the intensity level of PA of our participants was low and possibly too low to achieve health benefits. Unfortunately we did not have the availability of reference data for the level of intensity, but when we compare our results to Balemans et al., who used similar methodology, we see that wheelchair-using children with SB seem to show lower PA intensities than both typically developing children and ambulating children with CP (about 50% met the PA guideline) [24]. A recent meta-analysis in adults showed that high levels of moderate PA intensity attenuates increased risk of death associated with high sitting time [27]. Even though we do not have this evidence in children yet, these low levels of PA need our attention in pediatric rehabilitation practice.

As evidence has shown that PA during childhood tracks into adulthood, the challenge seems to be how to improve PA during early childhood [28]. There seems to be an opportunity by increasing active modes of transport and thus self-propelling the wheelchair or hand-biking, instead of being pushed by a person. When comparing wheeling and hand-biking of our participants to walking and biking in the reference group, we see that typically developing youth is more physically active in these activities than wheelchair-using youth with SB. There also seems to be an opportunity during weekends, as the participants seemed to be more sedentary and less physically active on a weekend day compared to a school day. The participants might have been fatigued after a school week and thus needing to rest. It might also be, however, that there are not enough possibilities to be physically active during weekends (f.e. playing adaptive sports or playing in the playground) or that there is not enough stimulation in the environment. Recent literature showed a variety of important facilitators and barriers (f.e. the lack of adaptive sports) when aiming to improve PA in youth with SB. The authors stated that we should focus on individual possibilities for that specific child and context, so applying an individual approach and not an ‘one-size fits all program’ [29, 30]. Future research may give insight in possible effective interventions, as evidence for improving PA in youth with physical disabilities is unfortunately very scarce [31].

Intensity of daily PA varied extensively between wheelchair-using participants, with e.g. wheeling and (hand)biking ranging from very light intensities to near to maximal. In general, activities as wheeling and (hand)biking can be adequate in achieving higher intensities. However, the differences we found underline the individual approach needed when aiming to improve PA. Interestingly, the variability from very light to near to maximal intensity was also found for sitting. This might be due to the fact that HR responses during exercise are slightly delayed and thus not fully in line with the activity that is performed [7]. For example, when a participant starts wheeling, it takes time for the HR to increase and to become in a steady state. Similarly, if a participant stops wheeling and holds his hands still (and thus sits according to the VitaMove), it takes time for the HR to recover and return to its resting rate. At the same time, we know HR can also be influenced by other factors than PA, e.g. stress or the use of caffeine, which we were not able to control for [7]. Our participants did not take any medication influencing the HR.

### Study limitations

To our knowledge, we were the first to measure PA objectively in wheelchair-using children. An important strength was that we used two valid objective devices simultaneously. By doing so, we were able to measure time spent sedentary and time spent physically active, as well as the intensity level. Moreover, it offered the unique possibility of combining these results into intensity during several activities.

Unfortunately, utilization of these objective devices also leads to a few limitations. We had about 35% of missing data, mostly because the devices did not function properly, but in some cases the minimum wear time was not met because children did not want to wear the devices anymore or because of skin irritation. When interpreting our results, one should keep in mind that they are based on 25–36 participants. Furthermore, the reference group for the VitaMove was rather small which may also have influenced the representativeness. One could think that wearing the devices might have affected PA negatively, but we did not find any reasons for that hypothesis, not in the activity diaries and also not after consulting parents.

A recent review describes that 7 monitoring days are ideal with a wear time of 10 h per day [32]. When looking at the amount of monitoring days, it was our clinical judgement that 3 monitoring days was maximal for these children because of feasibility. It is still very difficult to measure type of activity in wheelchair-using children, because the VitaMove is the only activity monitor that can measure type of activity validly in this population at this moment. Our participants had to wear three or even five devices and some children experienced this as a burden, which influences the feasibility negatively. When we analyzed the results of the two schooldays, we did not find significant differences between the two school days indicating that PA was similar on these school days. Because there seemed to be a difference between the school days and weekend days, we advise to measure at least a school day and a weekend day in this population. When looking at the minimum wear time, we chose a minimum wear time of 8 h instead of 10 h, because we also included young children. These young children go to bed early and also spent a large amount of time on personal and bowl and bladder care. During this time, they are not able to wear devices. Despite these limitations, we do believe our data adds to the current literature and helps to understand PA in this population.

Finally, the analysis and interpretation of daily activity data are quite time consuming. Because of this time consuming process, the use of daily activity monitoring is not feasible for clinical practice yet. Until now, the VitaMove is the only available valid device measuring type of activity in wheelchair-using children and thus very valuable, but it requires expensive equipment and special software. The Actiheart is less expensive and easier to use than the VitaMove, but analyzing several days of heartrate data still takes time. For clinical practice, measuring intensity of daily activities seems a good place to start for now when assessing PA in a wheelchair-using child. However, there is still a huge challenge in developing valid and reliable activity monitors that can easily be used in daily clinical practice in wheelchair-using youth, so clinicians will be able to measure PA individually in this population. It will support clinical reasoning and the development and evaluation of individually tailored interventions.

## Conclusions

Wheelchair-using youth with SB are substantially more sedentary and less physically active (both in type of activity and in intensity level) compared to typically developing peers. Furthermore, the physical activity levels on school days seem to be more favorable than the physical activity levels on a weekend day. We think that the low levels of PA need our attention in pediatric rehabilitation practice. The intensity of the different activities varied extensively between the participants, indicating the importance of individually tailored assessments and interventions.

## Abbreviations

PA: Physical activity
SB: Spina bifida
